# Five-year risk of end-stage renal disease among intensive care patients surviving dialysis-requiring acute kidney injury: a nationwide cohort study

**DOI:** 10.1186/cc12824

**Published:** 2013-07-22

**Authors:** Henrik Gammelager, Christian Fynbo Christiansen, Martin Berg Johansen, Else Tønnesen, Bente Jespersen, Henrik Toft Sørensen

**Affiliations:** 1 Department of Clinical Epidemiology, Aarhus University Hospital, Aarhus, Denmark; 2 Department of Anesthesiology and Intensive Care Medicine, Aarhus University Hospital, Aarhus, Denmark; 3 Department of Nephrology, Aarhus University Hospital, Aarhus, Denmark

**Keywords:** acute kidney injury, cohort studies, critical care, end-stage renal disease, intensive care units, prognosis, renal dialysis

## Abstract

**Introduction:**

Dialysis-requiring acute kidney injury (D-AKI) is common among intensive care unit (ICU) patients. However, follow-up data on the risk of end-stage renal disease (ESRD) among these patients remain sparse. We assessed the short-term and long-term risk of ESRD after D-AKI, compared it with the risk in other ICU patients, and examined the risk within subgroups of ICU patients.

**Methods:**

We used population-based medical registries to identify all adult patients admitted to an ICU in Denmark from 2005 through 2010, who survived for 90 days after ICU admission. D-AKI was defined as needing acute dialysis at or after ICU admission. Subsequent ESRD was defined as a need for chronic dialysis for more than 90 days or a kidney transplant. We computed the cumulative ESRD risk for patients with D-AKI and for other ICU patients, taking into account death as a competing risk, and computed hazard ratios (HRs) using a Cox model adjusted for potential confounders.

**Results:**

We identified 107,937 patients who survived for 90 days after ICU admission. Of these, 3,062 (2.8%) had an episode of D-AKI following ICU admission. The subsequent risk of ESRD up to 180 days after ICU admission was 8.5% for patients with D-AKI, compared with 0.1% for other ICU patients. This corresponds to an adjusted HR of 105.6 (95% confidence interval (CI): 78.1 to 142.9). Among patients who survived 180 days after ICU admission without developing ESRD (*n *= 103,996), the 181-day to 5-year ESRD risk was 3.8% for patients with D-AKI, compared with 0.3% for other ICU patients, corresponding to an adjusted HR of 6.2 (95% CI: 4.7 to 8.1). During the latter period, the impact of AKI was most pronounced in the youngest patients, aged 15 to 49 years (adjusted HR = 12.8, 95% CI: 6.5 to 25.4) and among patients without preexisting chronic kidney disease (adjusted HR = 11.9, 95% CI: 8.5 to 16.8).

**Conclusion:**

D-AKI is an important risk factor for ESRD for up to five years after ICU admission.

## Introduction

Acute kidney injury is a common organ dysfunction that may lead to or complicate intensive care unit (ICU) admission [1]. Among ICU patients, 4% to 6% have dialysis-requiring AKI (D-AKI) [2-4], which is associated with increased short-term and long-term mortality compared to ICU patients without this condition [5,6].

End-stage renal disease (ESRD), including need for chronic dialysis or kidney transplantation, is associated with considerable costs and impaired quality of life [7]. Studies of hospitalized patients found that D-AKI is an important risk factor for ESRD [8,9]. However, as AKI is often secondary to other diseases [1], its impact on subsequent ESRD may differ in ICU patients compared to other hospitalized patients in general and within subgroups of ICU patients. Knowledge of the long-term risk of ESRD after D-AKI in ICU patients, both overall and within subgroups, remains sparse, especially for patients who regain sufficient renal function to discontinue dialysis after an episode of D-AKI.

Previous studies have primarily reported dependency on dialysis at hospital discharge, or up to 180 days after initiating acute dialysis in the ICU [10-18]. However, few studies have followed patients beyond 180 days to examine the long-term ESRD risk after D-AKI in an ICU [3,11,19-21].

These studies are all limited by lack of a comparison cohort of ICU patients without D-AKI [3,11,19-21]. None examined potentially different impacts in subgroups of ICU patients [3,11,19-21], and only one ICU-based study reported the risk of ESRD among D-AKI patients who initially survived without developing ESRD [11].

We, therefore, conducted a nationwide cohort study among all ICU patients who survived 90 days or more after ICU admission, in order to examine the risk of ESRD among patients with D-AKI compared with other ICU patients. We examined short-term ESRD risk up to 180 days after ICU admission, and long-term ESRD risk from 181 days to 5 years for those who did not develop ESRD within the first 180 days after ICU admission. Thereby, we were able to both examine the short-term risk of ESRD after D-AKI, and the long-term risk in those patients who initially recovered sufficient renal function to become dialysis independent. In addition, we examined whether the impact of D-AKI on risk of ESRD varied across subgroups of ICU patients according to age, gender, chronic kidney disease, diabetes and surgical status.

## Methods

### Setting

We conducted this cohort study using prospectively collected data from medical registries in Denmark from 1 January 2005 to 31 December 2010 (Denmark had 5,411,405 inhabitants on 1 January 2005). The Danish National Health Service provides tax-supported health care to all Danish residents, including unfettered access to public hospitals. All intensive care and associated treatments are provided at public hospitals [22]. Denmark has 48 ICUs, including 37 multidisciplinary ICUs, four neurosurgical ICUs, three cardiothoracic ICUs, two multidisciplinary/cardiothoracic ICUs, one multidisciplinary/neurosurgical ICU, and onecardiac ICU.

In Denmark, unambiguous linkage of all registries is possible using a unique civil registration number assigned to all Danish residents by the Danish Civil Registration System (CRS). This registry contains complete information on migration, vital status and exact date of death for all residents [23].

### ICU patients

We used the Danish National Registry of Patients (DNRP) to identify all adult Danish residents (aged 15 years or older) with a first-time ICU admission from 1 January 2005 to 31 December 2010. Since 1977, it has been mandatory for hospitals in Denmark to report information on all in-hospital contacts to the DNRP. Since 1995, this registry has also included all emergency room and outpatient clinic visits. Variables in the registry include civil registration number, hospital and hospital department, date of hospital admission and discharge, emergency vs. planned hospital admission, surgical procedures, major treatments, and one primary diagnosis (the main reason for current hospitalization assigned by the discharging physician) and up to 19 secondary diagnoses. Since 1994, diagnoses have been coded according to the *International Classification of Diseases*, 10^th ^revision (ICD-10) [24]. Data on ICU admissions have been coded in the DNRP with a high level of accuracy since 2005 [25].

We used the primary ICD-10 diagnosis of the hospitalization to categorize patients into nine major disease groups: septicemia, other infectious disease, endocrine disease, cardiovascular disease, respiratory disease, gastrointestinal or liver disease, cancer, trauma or poisoning and other (please see Additional file 1 for the ICD-10 codes). In addition, we categorized patients into five groups according to surgical status: no surgery, acute cardiac surgery, acute non-cardiac surgery, elective cardiac surgery and elective non-cardiac surgery [26].

### Acute kidney injury

D-AKI was defined as the need for acute dialysis at or within 90 days after ICU admission, based on a Danish procedure code for acute dialysis in the DNRP. Acute dialysis includes dialysis and hemofiltration. All ICU patients not receiving acute dialysis were defined as 'other ICU patients'.

### End-stage renal disease

Data on ESRD were obtained from the Danish National Registry on Regular Dialysis and Transplantation (NRDT). The NRDT, established in 1990, contains highly valid information on all Danish residents with chronic kidney disease actively treated with either dialysis or kidney transplantation. Only patients with a permanent need of dialysis or need of a kidney transplant are included in the NRDT, thereby excluding patients with reversible kidney failure [27]. Registry data include date of first active treatment, treatment modality and underlying kidney disease. We defined date of ESRD diagnosis as 90 days after first active treatment registered in the NRDT, because chronic disease of the kidney is defined as impairment for more than 90 days [28]. Patients who died within 90 days after the first treatment were consequently not considered ESRD patients (*n *= 25). Patients with preexisting ESRD or any previous dialysis treatment were not included in the study.

### Covariates

Data on age, gender and comorbidity were obtained from the CRS and the DNRP. We obtained data on comorbidities that are associated with D-AKI and also are potential risk factors for ESRD. Using the DNRP, we identified all previous inpatient or outpatient clinic diagnoses up to five years before the current hospitalization. We thus included previous diagnoses of chronic kidney diseases, diabetes, hypertension, congestive heart failure, myocardial infarction, cerebrovascular disease, peripheral vascular disease and malignant neoplasms. (Please see Additional file 1 for relevant codes used in the current study).

### Statistical analyses

Patient characteristics, including demographic characteristics, comorbidity and information from the current hospitalization, were tabulated for ICU patients with and without D-AKI. We followed patients who survived the first 90 days after ICU admission until ESRD, death, emigration, five years from ICU admission or until 31 December 2011, whichever came first.

We plotted a 5-year cumulative risk curve for ESRD and calculated 90-day to 180-day, 181-day to 5-year, and overall 5-year risk of ESRD, using the cumulative risk method, which takes into account death as a competing risk [29]. We computed hazard ratios (HRs) for ESRD as a measure of relative risk, using a Cox proportional hazards regression model controlling for age group, gender, preexisting chronic kidney disease, diabetes, myocardial infarction, congestive heart failure, peripheral vascular disease, cerebrovascular disease, malignant neoplasms and surgical status. The assumptions of proportional hazards were checked graphically by log(-log) plots and found appropriate.

We examined the potentially different impact in subgroups of ICU patients, by stratifying the analyses by age groups, gender, presence chronic kidney disease, presence diabetes and surgical status. Analyses were performed using the statistical software package Stata version 11.1 (StataCorp LP, College Station, TX, USA).

All data were obtained from Danish registries, which are available to researchers, and their use does not require ethical approval or informed consent. The study was approved by the Danish Data Protection Agency (record number 2009-41-3987).

## Results

### Characteristics of the study population

The study population consisted of 107,937 adult ICU patients. Patients who died during the first 90 days after ICU admission (*n *= 33,367), and patients with preexisting ESRD or any previous dialysis treatment (*n *= 1,697) were not considered for the study. Total follow-up time was 230,278 person-years, with a median duration of 3.1 years (inter-quartile range (IQR): 1.6 to 4.8).

We found that 3,062 (2.8%) patients who survived for 90 days or more had an episode of D-AKI following ICU admission. Compared to other ICU patients, patients with D-AKI were slightly older (median age = 65 years (IQR: 55 to 73) vs. median age 62 = years, (IQR: 46 to 72)), more often male and, in general, with more preexisting comorbidity, in particularly chronic kidney disease, diabetes and hypertension. They also had longer hospital stays, were more often treated with mechanical ventilation or inotropes/vasopressors, and more often had a primary diagnosis of septicemia (Table 1).

**Table 1:** Characteristics by D-AKI status among 107,937 adult ICU patients, Denmark 2005 to 2010

	D-AKI^a^ *n *= 3,062	Other ICU patients^a^ *n *= 104,875
**Age, median (IQR), years**	65 (55, 73)	62 (46, 72)
**Gender**		
Female	1,116 (36.4)	45,440 (43.3)
Male	1,946 (63.6)	59,435 (56.7)
**Preexisting comorbidity **		
Chronic kidney disease^b^	325 (10.6)	1,961 (1.9)
Diabetes	547 (17.9)	8,715 (8.3)
Hypertension	748 (24.4)	16,218 (15.5)
Congestive heart failure	307 (10.0)	5,837 (5.6)
Myocardial infarction	147 (4.8)	4,848 (4.6)
Cerebrovascular disease	244 (8.0)	7,933 (7.6)
Peripheral vascular disease	286 (9.3)	6,517 (6.2)
Malignant neoplasm	329 (10.7)	12,402 (11.8)
**Primary diagnosis during current hospitalization**		
Septicemia	295 (9.6)	1,373 (1.3)
Other infectious diseases	300 (9.8)	8,087 (7.7)
Endocrinology diseases	86 (2.8)	2,126 (2.0)
Cardiovascular diseases	752 (24.6)	28,154 (26.9)
Respiratory diseases	298 (9.7)	7,502 (7.2)
Gastrointestinal or liver diseases	274 (8.9)	8,123 (7.7)
Cancer	195 (6.4)	13,321 (12.7)
Trauma or poisoning	250 (8.2)	18,427 (17.6)
Other	612 (20.0)	17,762 (16.9)
**Surgical status^c,d^**		
No surgery	1,250 (40.8)	38,406 (36.6)
Surgery		
Acute cardiac surgery	162 (5.3)	2,510 (2.4)
Acute non-cardiac surgery	1,126 (36.8)	30,559 (29.1)
Elective cardiac surgery	248 (8.1)	14,080 (13.4)
Elective non-cardiac surgery	276 (9.0)	19,320 (18.4)
**ICU treatments**		
Mechanical ventilation	2,320 (75.8)	33,742 (32.1)
Inotropes/vasopressors	2,244 (73.3)	26,705 (25.5)
**Length of admission, median (IQR)**		
In-hospital days	47 (26 to 84)	10 (5 to 20)
In-hospital days before ICU admission	1 (0 to 4)	1 (0 to 3)
In-hospital days after ICU admission	43 (24 to 80)	8 (4 to 16)

^a^Values are expressed as numbers (percentages) unless otherwise indicated.

^b^Patients with preexisting ESRD or who were previously treated with dialysis were not included in the current study.

^c^Surgical status identified by surgery at or up to seven days before ICU admission.

^d^Acute and elective status classified according to hospital admission type.

CI, confidence interval; D-AKI, dialysis-requiring acute kidney injury; ESRD, end-stage renal disease; ICU, intensive care unit; IQR, inter-quartile range

### Risk of end-stage renal disease

**Figure 1:**
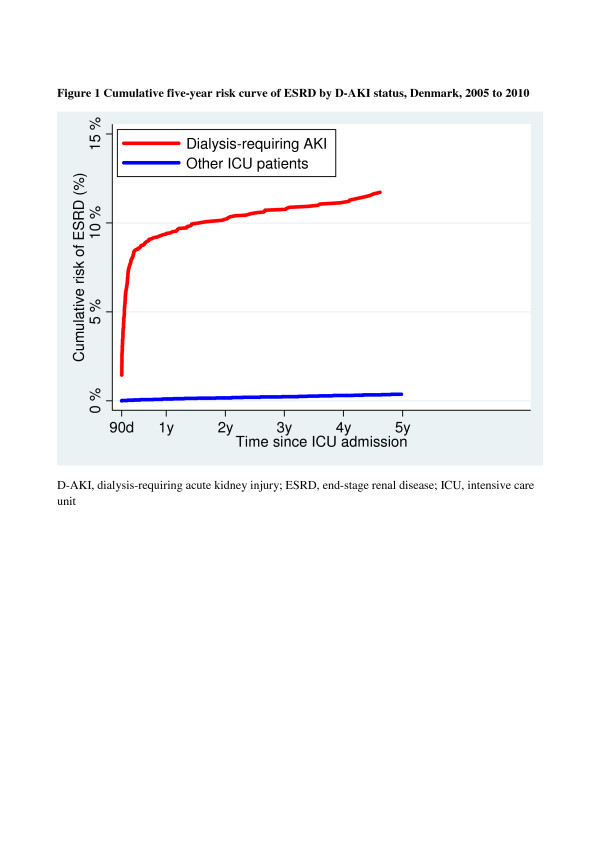
**Cumulative five-year risk curve of ESRD by D-AKI status, Denmark, 2005 to 2010**. AKI, acute kidney injury; ESRD, end-stage renal disease; ICU, intensive care unit

The five-year ESRD risk was 11.7% (95% CI: 10.5 to 13.0) for ICU patients with an episode of D-AKI, compared with 0.4% (95% CI: 0.3% to 0.4%) for other ICU patients (Figure 1).

#### Risk of ESRD within 90 to 180 Days

Out of the 3,062 ICU patients with D-AKI who survived for 90 days after ICU admission, 260 developed ESRD within 180 days following ICU admission (cumulative risk = 8.5% (95% CI: 7.5% to 9.5%)) compared with 57 patients out of 104,875 other ICU patients (cumulative risk = 0.1%, 95% CI: 0.0% to 0.1%). This corresponds to an unadjusted HR for ESRD of 165.9 (95% CI: 124.6 to 221.0) for D-AKI patients compared with other ICU patients. After adjusting for potential confounders, with chronic kidney disease being the most important, the adjusted HR was 105.6 (95% CI: 78.1 to 142.9) (Table 2).

**Table 2:** Cumulative risk and hazard ratios of ESRD for ICU patients with D-AKI compared to other ICU patients

Follow up/cohort	Total (n)	ESRD (n)	Cumulative risk % (95% CI)	Unadjusted HR (95% CI)	Adjusted HR^a^ (95% CI)
**90 to 180 days**					
D-AKI	3,062	260	8.5 (7.5- 9.5)	165.9 (124.6 to 221.0)	105.6 (78.1 - 142.9)
Other ICU patients	104,875	57	0.1 (0.0 - 0.1)	1 (reference)	1 (reference)
**181 days to 5 years**					
D-AKI	2,579	76	3.8 (3.0 - 4.8)	13.5 (10.5 - 17.5)	6.2 (4.7 - 8.1)
Other ICU patients	101,417	249	0.3 (0.3 - 0.4)	1 (reference)	1 (reference)

^a^Adjusted for age group, gender, chronic kidney disease, diabetes, hypertension, congestive heart failure, myocardial infarction, peripheral vascular disease, cerebrovascular disease, cancer and surgical status.

CI, confidence interval; D-AKI, dialysis-requiring acute kidney injury; ESRD, end-stage renal disease; HR, hazard ratio; ICU, intensive care unit; n, number

#### Risk of ESRD within 181 days to 5 years

Among ICU patients who survived 180 days after ICU admission without developing ESRD, the 181-day to 5-year ESRD risk for patients with D-AKI was 3.8% (95% CI: 3.0% to 4.8%), compared with 0.3% (95% CI: 0.3% to 0.4%) for other ICU patients. This corresponds to an unadjusted HR of 13.5 (95% CI: 10.5 to 17.5) and an adjusted HR of 6.2 (95% CI: 4.7 to 8.1) (Table 2). Again, chronic kidney disease was the most important confounder in the adjusted model.

#### Subgroup analyses

The association between D-AKI and ESRD was evident within all subgroups of ICU patients in the 90- to 180-day period (Table 3). Within the 181-day to 5-year follow-up period, we found that the relative impact of D-AKI on risk of ESRD was higher for patients without preexisting chronic kidney disease (adjusted HR = 11.9 (95% CI: 8.5 to 16.8)) than for patients with preexisting chronic kidney disease (adjusted HR = 2.8 (95% CI: 1.8 to 4.3)) due to a high risk of ESRD even without AKI (cumulative risk = 7.2% (95% CI: 5.9% to 8.8%)). Nonetheless, the absolute risk difference between D-AKI patients and other ICU patients was remained higher for patients with preexisting kidney disease compared to patients without this diagnosis (Table 4). The relative impact of D-AKI on risk of future ESRD was also most pronounced in the youngest age group, in females and among elective surgical patients, but similar among diabetic and non-diabetic patients (Table 4).

**Table 3:** ESRD risk and adjusted HR between 90 and 180 days after ICU admission by D-AKI status

	D-AKI			Other ICU patients _			Adjusted HR (95% CI)^a^
	Total (n)	ESRD (n)	Cumulative risk % (95% CI)	Total (n)	ESRD (n)	Cumulative risk % (95% CI)	
**Age, years**							
15 to 49	533	30	5.6 (3.9 - 7.8)	30,629	9	0.03 (0.01 - 0.06)	121.6 (54.2 - 272.9)
50 to 69	1,444	120	8.3 (7.0 - 9.8)	42,078	26	0.06 (0.04 - 0.09)	91.6 (58.7 -143.0)
≥70	1,085	110	10.1 (8.4 - 12.0)	32,159	22	0.07 (0.04 - 0.10)	109.0 (67.6 -175.6)
**Gender**							
Female	1,116	89	8.3 (6.8 - 9.9)	45,441	21	0.05 (0.03 - 0.07)	117.3 (70.8 - 194.5)
Male	1,946	171	8.4 (6.9 - 10.1)	59,435	36	0.06 (0.04 - 0.08)	98.7 (67.7 - 143.9)
**Chronic kidney disease**							
Yes	325	90	27.7 (22.9 - 32.6)	1,961	38	1.94 (1.40 - 2.62)	17.4 (11.8 - 25.7)
No	2,737	170	6.2 (5.3 - 7.2)	102,914	19	0.02 (0.01 - 0.03)	313.0 (193.4 - 506.4)
**Diabetes**							
Yes	547	62	11.3 (8.8 - 14.2)	8,715	15	0.17 (0.10 -0.28)	52.4 (29.0 - 94.8)
No	2,515	198	7.9 (6.8 - 9.0)	96,160	42	0.04 (0.03 - 0.06)	122.6 (86.5 - 173.8)
**Surgical status**							
No surgery	1,250	129	10.3 (8.7 - 12.1)	38,406	32	0.08 (0.06 - 0.12)	83.1 (55.2 - 125.0)
Surgery							
Acute non-cardiac surgery	1,126	83	7.3 (5.9 - 9.0)	30,559	11	0.04 (0.02 - 0.06)	145.3 (76.4 - 276.2)
Acute cardiac surgery	162	8	4.9 (2.3 - 9.0)	2,510	4	0.16 (0.06 - 0.40)	25.2 (6.7 to 94.9)
Elective non-cardiac surgery	276	28	10.1 (6.9 - 14.0)	19,320	7	0.04 (0.02 - 0.07)	218.9 (91.1 - 521.1)
Elective cardiac surgery	248	12	4.8 (2.6 - 8.0)	14,080	3	0.02 (0.00 - 0.06)	136.2 (33.1 - 561.3)

^a^Compared to ICU patents not treated with dialysis within same subgroup and adjusted for age group, gender, chronic kidney disease, diabetes, hypertension, congestive heart failure, myocardial infarction, peripheral vascular disease, cerebrovascular disease, cancer and surgical status.

CI, confidence interval; D-AKI, dialysis-requiring acute kidney injury; ESRD, end-stage renal disease; HR, hazard ratio; ICU, intensive care unit; n, number

**Table 4:** ESRD risk and adjusted HR between 181 days and five years after ICU by D-AKI status

	D-AKI			Other ICU patients			Adjusted HR (95% CI)^a ^
	Total (n)	ESRD (n)	Cumulative risk % (95% CI)	Total (n)	ESRD (n)	Cumulative risk % (95% CI)	
**Age, y**							
15 to 49	484	14	3.4 (1.9 - 5.5)	30,299	38	0.18 (0.13 - 0.24)	12.8 (6.5 - 25.4)
50 to 69	1,227	33	3.9 (2.6 - 5.4)	40,750	129	0.44 (0.37 - 0.53)	5.3 (3.6 - 8.0)
≥70	864	29	4.0 (2.7 - 5.7)	30,330	82	0.35 (0.28 - 0.43)	6.9 (4.4 - 10.9)
**Gender**							
Female	952	34	5.0 (3.2 - 7.5)	43,955	99	0.30 (0.24 - 0.37)	7.3 (4.8 - 11.1)
Male	1,627	42	3.3 (2.3 - 4.6)	57,462	150	0.36 (0.30 - 0.42)	5.5 (3.9 - 8.0)
**Chronic kidney disease**							
Yes	218	28	18.4 (12.2 - 25.6)	1,795	102	7.23 (5.94 - 8.81)	2.8 (1.8 - 4.3)
No	2,361	48	2.7 (2.0 - 3.6)	99,622	147	0.21 (0.18 - 0.25)	11.9 (8.5 - 16.8)
**Diabetes**							
Yes	434	29	10.3 (6.7 - 14.7)	8,232	83	1.51 (1.20 - 1.88)	6.0 (3.9 - 9.4)
No	2,145	47	2.7 (2.0 - 3.6)	93,102	166	0.23 (0.20 - 0.27)	6.7 (4.7 - 9.4)
**Surgical status**							
No surgery	1,033	33	4.3 (2.9 - 6.1)	36,986	100	0.35 (0.28 - 0.42)	6.0 (4.0 - 9.2)
Surgery							
Acute non-cardiac surgery	960	23	3.0 (1.9 - 4.5)	29,341	73	0.35 (0.27 - 0.44)	5.8 (3.5 - 9.5)
Acute cardiac surgery	143	1	1.0 (0.1 - 5.9)	2,478	5	0.27 (0.10 - 0.62)	3.9 (0.4 - 39.9)
Elective non-cardiac surgery	226	5	2.8 (1.0 - 6.1)	18,640	34	0.25 (0.17 - 0.36)	8.7 (3.2 - 23.3)
Elective cardiac surgery	217	14	8.2 (4.6 - 13.2)	13,972	37	0.39 (0.28 - 0.54)	10.8 (5.3 - 22.3)

^a^Compared to ICU patents not treated with dialysis within the same subgroup and adjusted for age group, gender, chronic kidney disease, diabetes, hypertension, congestive heart failure, myocardial infarction, peripheral vascular disease, cerebrovascular disease, cancer and surgical status.

CI, confidence interval; D-AKI, dialysis requiring acute kidney injury; ESRD, end-stage renal disease; HR, hazard ratio; ICU, intensive care unit; n, number

## Discussion

### Key results

This nationwide population-based cohort study extends current knowledge by examining and comparing both short-term and long-term risk of ESRD among ICU patients with and without an episode of D-AKI. Among ICU patients who survived for 90 days after ICU admission, we found the five-year absolute risk of ESRD to be more than 10% in ICU patients with D-AKI compared with a risk of less than 0.5% for other ICU patients. Even among patients who survived more than 180 days after ICU admission without having ESRD, the subsequent ESRD risk was six-fold higher for up to five years among ICU patients with an episode of D-AKI compared to other ICU patients. The relative impact of D-AKI on ESRD risk was less pronounced for patients with chronic kidney disease, due to their high risk of ESRD even without D-AKI.

### Existing studies

In line with our observations on the short-term risk of ESRD, a Swedish cohort study reported that 9.4% (104/1,102) of ICU patients with D-AKI, who were alive 90 days after commencement of acute dialysis, had started active treatment for ESRD [11]. Slightly lower estimates were reported by Bellomo *et al*. from their randomized trial that examined the optimal intensity of dialysis. They found that 5.6% (45/810) of patients with D-AKI still were dependent on dialysis 90 days after initiating acute dialysis [12]. However, in contrast, Cartin-Ceba *et al*. found that as many as 37.0% (282/784) of ICU patients who survived and were discharged from hospital needed dialysis for more than 90 days [13]. Furthermore, small studies including between 17 and 137 surviving ICU patients with D-AKI in various ICU settings have reported that between 4.2% and 28.9% are still dependent on dialysis 90 or 180 days after initiating acute dialysis [3,14-18]. This large variation may be explained primarily by differences in ICU populations with different baseline renal function.

Our estimates of long-term ESRD risk are also consistent with the Swedish study by Bell *et al. *[11]. They found that 3.4% (34/998) of ICU patients with D-AKI developed ESRD in the period from 90 days up to seven years after initiating dialysis [11]. Our results are also consistent with both a Canadian study and a US study of hospitalized patients, which found an increased risk of ESRD in patients requiring acute dialysis who initially recovered enough renal function to discontinue dialysis [8,9]. The Canadian study by Wald *et al*. reported an adjusted HR of 3.23 (95% CI: 2.70 to 3.86) for ESRD after D-AKI among hospitalized patients who did not develop ESRD in the first month after hospital discharge, compared to a matched cohort of patients without D-AKI [8]. Results were similar for a subgroup analysis of mechanically ventilated patients (*n *= 1,716) used as a surrogate for ICU admission. The US study by Hsu *et al*. examined patients with known chronic kidney disease (estimated glomerular filtration rate of below 45 ml/minute per 1.73m^2^) with D-AKI during hospitalization who did not develop ESRD within the first month after hospital discharge. The study reported an adjusted HR of 1.47 (95% CI: 0.95 to 2.28) for ESRD [9]. However, studies including between 39 and 105 surviving ICU patients in different ICU settings have reported the proportion still dependent on dialysis to be 13.3% (8/60), 21.8% (19/87) and 33.3% (13/39) one year after initiating acute dialysis [3,19,21], and 1.7% (1/60) three years after initiating acute dialysis[20].

We did not have data on the mechanism of the potential association between D-AKI and subsequent ESRD. We speculate that some patients may never recover normal kidney function after D-AKI, as observed among patients who developed ESRD in the 90- to 180-day period. A recent study showed that even patients who recover normal kidney function after AKI as assessed by serum creatinine measurements are at considerably increased risk of subsequent ESRD [30]. However, these patients may have residual renal function impairment, which is not detectable by elevated plasma creatinine [31].

### Clinical perspectives

Our study suggests that D-AKI among ICU patients is a strong risk factor for ESRD for up to five years after ICU admission. Thus, there may be a need for systematic post-discharge follow-up of ICU patients with an episode of D-AKI, in order to avoid further kidney damage, and for development of prophylactic strategies.

### Strengths and limitations

The strengths of our study include access to a well-defined population with uniform access to health care, use of high quality nationwide medical databases, and virtually complete follow-up data. This minimized selection and information biases.

Still, some limitations should be considered when interpreting our results. First, we lacked detailed nationwide data on kidney function, such as serum creatinine measurements to estimate glomerular filtration rates (eGFR). Thus, we were unable to identify and stage pre-existing chronic kidney disease by eGFR level. Neither were we able to assess the subsequent risk of kidney dysfunction less severe than ESRD. However, even if we had creatinine measurements, the availability of more measurements in patients with a D-AKI history is likely to introduce bias. Second, we lacked access to detailed data on type or intensity of dialysis performed in the ICUs. Therefore, we were unable to examine the association between type or intensity of acute dialysis and ESRD. Third, despite adjustment for potential confounders, including age group, comorbidity and surgical status, we cannot rule out unmeasured and residual confounding, for example, by severity of preexisting chronic kidney disease.

## Conclusions

We found that more than one out of 10 ICU patients who survived 90 days after ICU admission with D-AKI developed ESRD during the five years of follow-up, compared with less than one out of 200 in other ICU patients. Thus, an episode of D-AKI among ICU patients is an important risk factor for subsequent ESRD up to five years after ICU admission. While the increased risk compared with other ICU patients was evident within all subgroups of ICU patients, it was highest within subgroups with low baseline risk of ESRD, such as young patients and patients without chronic kidney disease.

## Key messages

• One out of 10 ICU patients surviving the first 90 days of D-AKI developed ESRD during the five years of follow-up.

• D-AKI is an important risk factor for ESRD, even among patients who initially recovered sufficient kidney function to discontinue dialysis.

• The relative risk of ESRD was highest within subgroups with low baseline risk of ESRD, such as young patients and patients without chronic kidney disease.

## Abbreviations

D-AKI: dialysis-requiring acute kidney injury; CI: confidence interval; CRS: Danish Civil Registration System; DNRP: Danish National Registry of Patients; eGFR: estimated glomerular filtration rates; ESRD: end-stage renal disease; HR: hazard ratio; ICU: intensive care unit; ICD-10: International Classification of Diseases: 10^th ^revision; IQR: inter-quartile range; NRDT: Danish National Registry on Regular Dialysis and Transplantation

## Competing interests

The authors declare that they have no competing interests.

## Authors' contributions

HG, CFC and HTS conceived the study idea. HG, CFC, MBJ, BJ and HTS designed the study. MBJ and HTS collected the data. HG and MBJ performed the statistical analyses. HG and CFC reviewed the literature. HG wrote the first draft. HG, CFC, MBJ, ET, BJ and HTS interpreted the findings. All authors critically reviewed and edited the manuscript and approved the final version.

## Supplementary Material

Additional File 1 Relevant codes used in the current study Click here for file
