# Supplementary material for: Five-year risk of end-stage renal disease among intensive care patients surviving dialysis-requiring acute kidney injury: a nationwide cohort study
Source: Crit Care. 2013 Jul 22;17(4):R145. doi: 10.1186/cc12824 (PMC4055988; doi:10.1186/cc12824)
Supplement: Additional File 1 — Relevant codes used in the current study [file cc12824-S1.DOCX]

| **Relevant codes used in the current study** | |
| --- | --- |
| **Description** | **Codes** |
| **ICU admission^a^** |  |
| Intensive observation | NABE |
| Intensive therapy | NABB |
| **Treatments^a^** |  |
| **Acute dialysis** | **BJFD0** |
| Mechanical ventilation | BGDA0 |
| Treatment with inotropes or vasopressors | BFHC92, BFHC93, BFHC95 |
| **Primary diagnosis during current hospitalization^b^** |  |
| Septicemia | A02.1, A22.7, A26.7, A28.2B, A32.7, A39.2, A40-A41, A.42.7, A54.8G, B37.7, J95.0A, O08.0S, O08.0T, O08.0U, O08.0V,O08.0Y, O85.9, R57.2, T80.2D, T80.2E, T80.2F, T81.4D, T88.0A |
| Other infectious diseases | A00–B99 (except: A02.1, A22.7, A26.7, A28.2B, A32.7, A39.2, A40-A41, A.42.7, A54.8G, B37.7), G00–G07, I00–I02, I30.1, I32.0, I33, I38, I40.0, J00–J06, J36, J39.0, J10–J22, J85.1, J86, K35, K37, K57.0, K57.2, K57.4, K57.8, K61, K63.0, K65.0, K65.9, K67, K75.0, K75.1, K80.0, K80.3, K80.4, K81.0, K81.9, K83.0, L00–L03, L05–L08, M00, M01, M86, N10, N12, N15.1, N30, N39.0, N41, N45, N70–N77 |
| Cancer | C00–D89 |
| Endocrinological diseases | E00–E90 |
| Cardiovascular diseases | I00–I99 without I00–I02, I30.1, I32.0, I33, I38, I40.0 |
| Respiratory diseases | J00–J99 without J00–J06, J10–J22, J36, J39.0, J85.1, J86, J95.0A |
| Gastrointestinal or liver diseases | K00–K99 without K35, K37, K57.0, K57.2, K57.4, K57.8, K61, K63.0, K65.0, K65.9, K67, K75.0, K75.1, K80.0, K80.3, K80.4, K81.0, K81.9, K83.0. |
| Trauma or poisoning | S00–S99, T00–T97 without T80.2D, T80.2E, T80.2F, T81.4D, T88.0A |
| Others | All codes not included in other categories |
| **Preexisting morbidity^b^** |  |
| Chronic kidney disease | N00–N08, N11, N14–N16, N18–N19, N26–N27, N28.0, N39.1, E10.2, E11.2, E14.2, I12.0, I13.1, I13.2, I15.0, I15.1 |
| Diabetes mellitus | E10–E11 |
| Hypertension | I10–I15 |
| Congestive heart failure | I50.x, I11.0, I13.0, I13.2 |
| Myocardial infarction | I21, I22, I23 |
| Peripheral vascular disease | I70, I71, I72, I73, I74, I77 |
| Cerebrovascular disease | I60–I69, G4, G46 |
| Malignant neoplasm | C00–C96 |
| ^a^ Danish treatment codes in the Danish National Registry of Patients  ^b^ International Classification of Diseases, 10th revision codes in the Danish National Registry of Patients | |

**Additional file 1**
